# Translational Studies on Anti-Atrial Fibrillatory Action of Oseltamivir by its *in vivo* and *in vitro* Electropharmacological Analyses

**DOI:** 10.3389/fphar.2021.593021

**Published:** 2021-04-29

**Authors:** Ryuichi Kambayashi, Hiroko Izumi-Nakaseko, Ai Goto, Kazuya Tsurudome, Hironori Ohshiro, Taku Izumi, Mihoko Hagiwara-Nagasawa, Koki Chiba, Ryota Nishiyama, Satomi Oyama, Yoshio Nunoi, Yoshinori Takei, Akio Matsumoto, Atsushi Sugiyama

**Affiliations:** ^1^ Department of Pharmacology, Faculty of Medicine, Toho University, Tokyo, Japan; ^2^ Sophion Bioscience K.K., Saitama, Japan; ^3^ Drug Research Department, TOA EIYO LTD., Fukushima, Japan; ^4^ Department of Translational Research and Cellular Therapeutics, Faculty of Medicine, Toho University, Tokyo, Japan; ^5^ Department of Aging Pharmacology, Faculty of Medicine, Toho University, Tokyo, Japan; ^6^ Yamanashi Research Center of Clinical Pharmacology, Yamanashi, Japan

**Keywords:** oseltamivir, pilsicainide, persistent atrial fibrillation, patch clamp assay, IK,ACh, dogs, ECG, safety pharmacology

## Abstract

Oseltamivir has been shown to prolong the atrial conduction time and effective refractory period, and to suppress the onset of burst pacing-induced atrial fibrillation *in vitro*. To better predict its potential clinical benefit as an anti-atrial fibrillatory drug, we performed translational studies by assessing *in vivo* anti-atrial fibrillatory effect along with *in vivo* and *in vitro* electropharmacological analyses. Oseltamivir in intravenous doses of 3 (n = 6) and 30 mg/kg (n = 7) was administered in conscious state to the persistent atrial fibrillation model dogs to confirm its anti-atrial fibrillatory action. The model was prepared by tachypacing to the atria of chronic atrioventricular block dogs for > 6 weeks. Next, oseltamivir in doses of 0.3, 3 and 30 mg/kg was intravenously administered to the halothane-anesthetized intact dogs to analyze its *in vivo* electrophysiological actions (n = 4). Finally, its *in vitro* effects of 10–1,000 μM on I_K,ACh_, I_Kur_, I_Kr_, I_Na_ and I_CaL_ were analyzed by using cell lines stably expressing Kir3.1/3.4, K_V_1.5, hERG, Na_V_1.5 or Ca_V_1.2, respectively (n = 3 for I_K,ACh_ and I_Kr_ or n = 6 for I_Kr_, I_Na_ and I_CaL_). Oseltamivir in doses of 3 and 30 mg/kg terminated the atrial fibrillation in 1 out of 6 and in 6 out of 7 atrial fibrillation model dogs, respectively without inducing any lethal ventricular arrhythmia. Its 3 and 30 mg/kg delayed inter-atrial conduction in a frequency-dependent manner, whereas they prolonged atrial effective refractory period in a reverse frequency-dependent manner in the intact dogs. The current assay indicated that IC_50_ values for I_K,ACh_ and I_Kr_ were 160 and 231 μM, respectively, but 1,000 µM inhibited I_Na_, I_CaL_ and I_Kur_ by 22, 19 and 13%, respectively. The extent of I_Na_ blockade was enhanced at faster beating rate and more depolarized resting membrane potential. Oseltamivir effectively terminated the persistent atrial fibrillation, which may be largely due to the prolongation of the atrial effective refractory period and inter-atrial conduction time induced by I_K,ACh_ and I_Kr_ inhibitions along with I_Na_ suppression. Thus, oseltamivir can exert a powerful anti-atrial fibrillatory action through its ideal multi-channel blocking property; and oseltamivir would become a promising seed compound for developing efficacious and safe anti-atrial fibrillatory drugs.

## Introduction

Since existing anti-atrial fibrillation (AF) drugs have been known to block both atrial and ventricular ionic channels, adverse effects were largely induced by their ionic channel blockade in the ventricle, which may often blunt their therapeutic benefits for the atrial arrhythmias (Geng et al., 2020). The ideal anti-AF drug should selectively improve the pathophysiology in the atria without affecting the electrophysiological profile of the ventricles.

Oseltamivir is a pro-drug of oseltamivir carboxylate, which has been used for treating patients with influenza virus infection (McClellan and Perry, 2001). We have demonstrated that 30 mg/kg, i.v. of oseltamivir delayed the atrial and ventricular conductions as well as ventricular repolarization in halothane-anesthetized dogs (Kitahara et al., 2013), that 3 and 30 mg/kg, i.v. of oseltamivir did not induce torsade de pointes in chronic atrioventricular block dogs (Nakamura et al., 2016), and that 100 μM of oseltamivir prolonged the atrial conduction time as well as the effective refractory period in the isolated guinea pig hearts (Takahara et al., 2013). Moreover, in a previous study using the Langendorff-perfused rabbit hearts, 100 μM of oseltamivir suppressed the onset of AF induced by burst pacing with ACh and isoproterenol pretreatment (Frommeyer et al., 2017). However, those known basic findings (Kitahara et al., 2013; Takahara et al., 2013; Nakamura et al., 2016; Frommeyer et al., 2017) would not be enough to make the clinical development of oseltamivir as an anti-AF drug valid.

In order to facilitate its clinical application, we performed the following translational studies. Initially, we assessed antiarrhythmic effects of oseltamivir on persistent AF model dogs, which was prepared by tachypacing to the atria of chronic atrioventricular block dogs for > 6 weeks, inducing structural and electrical remodeling that enables to maintain AF (Wijffels et al., 1995; Gaspo et al., 1997). Next, we examined electrophysiological effects of oseltamivir on the atrium of halothane-anesthetized intact dogs, which have been demonstrated to be able to mimic the drug-induced electropharmacological responses in human subjects (Sugiyama, 2008). These *in vivo* results were compared with those of clinically available class Ic drug pilsicainide, since pilsicainide as well as flecainide are recommended for treatment of AF in JCS/JHRS 2020 Guideline on Pharmacotherapy of Cardiac Arrhythmias in Japan (Plosker, 2010; Ono et al., 2020). Finally, using patch clamp assay, we studied the effects of oseltamivir on each of I_K,ACh_, I_Kur_, I_Kr_, I_Na_ and I_CaL_ to clarify which combination of the inhibitory action may play a pivotal role for suppressing AF.

## Materials and Methods

Experiments were performed by using 29 beagle dogs of either sex, aging 1–3 years and weighing approximately 10 kg, which were obtained through Kitayama Labes Co., Ltd. (Nagano, Japan). All experiments were reviewed and approved by Animal Care and User Committee of Yamanashi Research Center of Clinical Pharmacology (No.2005-16) and Toho University (No.17-52-324), and performed according to the Guideline for the Care and Use of Laboratory Animals of University of Yamanashi and Toho University, and ARRIVE guidelines (Kilkenny et al., 2010; McGrath et al., 2010). Randomization and allocation concealment of the animals were performed for each of the *in vivo* experiments. Data were analyzed under the open-label for all experiments, which were confirmed once more by another evaluator to secure their reliability. Animals showing the heart rate of > 200 bpm or < 50 bpm, mean blood pressure of > 150 mmHg or < 50 mmHg, and/or electrocardiogram of other than normal sinus rhythm after the initial induction of anesthesia were excluded from the studies.

### Experiment 1 (Exp. 1): Cardioversion Experiments

#### Surgical Preparation

After the animals (n = 21) were anesthetized with thiopental sodium (30 mg/kg, i.v.), catheter electrodes (Cordis-Webster Inc. CA, United States) was positioned at the compact atrioventricular node. The radiofrequency energy of 20 W was delivered for 10 s from the tip electrode to develop the complete atrioventricular block. More than 1 month later, the dogs were initially anesthetized pentobarbital sodium (30 mg/kg, i.v.) (Sugiyama, 2008). After intubation, anesthesia was maintained by isoflurane inhalation (1.5% v/v) vaporized in oxygen with a volume-limited ventilator. Tidal volume and respiratory rate were set at 20 ml/kg and 15 breaths/min, respectively. Initially, the skin of the gluteal region was incised to make a subcutaneous pocket for implanting the pacemaker. Next, the left fourth intercostal thoracotomy was performed and the pericardium was incised to expose the atrium. Two sets of the electrode of temporary pacing lead (Streamline atrial 6492; Medtronic Inc. MN, United States) were sutured onto the Bachmann bundle while lifting the left atrial appendage. The pericardial incision was fixed, and the thorax was closed. The pacing leads were connected to the rapid pulse-generating pacemaker (TNT-002AL; Taisho Biomed Instruments Co., Ltd. Osaka, Japan) placed at the gluteal region through the subcutaneous tunnel. The incision at the gluteal region was sutured carefully.

#### Induction of Persistent AF

More than 4 weeks after implanting a pacemaker, the atria were electrically driven at 400 bpm for 2 weeks. Then, the pacing rate was increased to 600 bpm to promote the atrial electrical remodeling. When the atrial pacing could not achieve 1:1 atrial capture at 600 bpm, the atrial pacing was maintained at 400–500 bpm until the atria could be electrically paced at 600 bpm. After confirming the onset of AF, the pacing was maintained at 600 bpm for 2 weeks. Then, the atrial pacing was stopped under the monitoring of Holter electrocardiogram (QR2100; Fukuda M-E Kogyo Co., Ltd. Tokyo, Japan). The duration of AF was assessed using the Holter analysis system (HS1000; Fukuda M-E Kogyo Co., Ltd.). When the AF persisted > 24 h, the dog was used as the persistent AF model animal to assess the anti-AF effect of drugs; whereas if it was terminated before 24 h, the pacing was restarted at 600 bpm for additional 1 week until AF persisted for > 24 h. In our preliminary study, once AF persisted for > 24 h, it did for > 1 week.

#### Experimental Protocol

The atria were electrically driven at 600 bpm for another one week. Under the monitoring of Holter electrocardiogram, the atrial pacing was stopped without anesthesia. Approximately 2 h after the termination of the atrial pacing, 3 (n = 6) and 30 mg/kg (n = 7) of oseltamivir as well as 3 mg/kg (n = 8) of pilsicainide hydrochloride hydrate were intravenously administered over 10 min.

### Experiment 2 (Exp. 2): Electrophysiological Studies

#### Surgical Preparation

The dogs were initially anesthetized with thiopental sodium (30 mg/kg, i.v.). After intubation, anesthesia was maintained by halothane inhalation (1% v/v) vaporized in oxygen with a volume-limited ventilator. Tidal volume and respiratory rate were set at 20 ml/kg and 15 breaths/min, respectively. The surface lead II electrocardiogram was continuously monitored, whereas the arterial blood pressure was measured through an indwelling needle placed at the right femoral artery.

Three sets of standard 6-French quad-polar electrodes catheter (Cordis-Webster Inc.) were used. The first one was positioned at the high right atrium through the right femoral vein to electrically pace the sinus nodal area and to simultaneously obtain the right atrial electrogram. The second one was positioned in the esophagus via the oral cavity to record the left atrial electrogram. The third one was positioned at the endocardium of the right ventricle through the left femoral vein to electrically pace the right ventricle.

#### Measurement of Electrophysiological Variables

The lead II electrocardiogram was obtained from the limb electrodes. The PR interval, QRS width, QT interval and P-wave duration were measured, and the QT interval was corrected with Van de Water’s formula: QTc = QT−0.087 × (RR−1,000) with RR given in ms, which was developed for anesthetized dogs (Van de Water et al., 1989). In addition, the J-T_peak_ and T_peak_-T_end_ were separately measured. The J-T_peak_ was corrected for heart rate with a coefficient as previously described (J-T_peak_c = J-T_peak_/RR^0.58^ with RR given in seconds) (Johannesen et al., 2014). Correction was not made on the T_peak_-T_end_, since previous QT studies have shown that the T_peak_-T_end_ exhibited minimal heart rate dependency at resting heart rate (Johannesen et al., 2014). The heart was electrically driven with the cardiac stimulator (SEC-3102; Nihon Kohden Corporation, Tokyo, Japan) through pacing electrodes of the catheters placed at the sinus nodal area or at the right ventricle. The stimulation pulses were set rectangular in shape, consisting of 1–2 V amplitude (about twice the threshold voltage) and 1 ms duration. The inter-atrial conduction time (IACT) was defined as an interval between the right and left atrial electrograms, which was measured during spontaneous sinus rhythm (IACT_(sinus)_) and at pacing cycle lengths of 400 ms (IACT_(CL400)_), 300 ms (IACT_(CL300)_) and 200 ms (IACT_(CL200)_). The atrial (AERP) and ventricular effective refractory period (VERP) were assessed with programmed electrical stimulation on the sinus nodal area and the right ventricle, respectively. The pacing protocol consisted of five beats of basal stimuli in cycle lengths of 400 ms (AERP_(CL400)_), 300 ms (AERP_(CL300)_) and 200 ms (AERP_(CL200)_) for AERP, and 400 ms (VERP_(CL400)_) for VERP followed by an extra stimulus of various coupling intervals. The coupling interval was shortened in 5 ms decrements until the additional stimulus could no longer elicit a response. AERP and VERP were defined as the shortest coupling interval that can evoke stimulus response.

#### Experimental Protocol

The right and left atrial electrograms, electrocardiogram and arterial blood pressure were monitored with a polygraph system (RM-6000; Nihon Kohden Corporation) and analyzed with real-time, fully automatic data analysis system (WinVAS3 ver. 1.1R24; Physio-Tech Co., Ltd. Tokyo, Japan). Each measurement of arterial pressure, electrocardiographic variables and IACT adopted mean of three recordings of consecutive complexes.

The electropharmacological variables were assessed in the following order. First, the right and left atrial electrograms, electrocardiogram and arterial blood pressure were recorded under the spontaneous sinus rhythm. Second, the sinus nodal area was paced at cycle lengths of 400, 300 and 200 ms to measure IACT. Third, AERP was assessed at basic pacing cycle lengths of 400, 300 and 200 ms. Fourth, VERP was measured at a basic pacing cycle length of 400 ms.

After the basal assessment, 0.3 mg/kg of oseltamivir was intravenously infused over 10 min, and each variable was assessed at 5, 10, 15, 20 and 30 min after the start of its administration (n = 4). Then, 3 mg/kg of oseltamivir was intravenously infused over 10 min, and each variable was assessed in the same manner. Finally, 30 mg/kg of oseltamivir was intravenously infused over 10 min, and each variable was assessed at 5, 10, 15, 20, 30, 45 and 60 min after the start of its administration. On the other hand, as another series of experiments, cardiovascular effects of 1 and 3 mg/kg of pilsicainide hydrochloride hydrate were assessed in the same manner (n = 4) as those for the middle and high doses of oseltamivir, respectively. We previously confirmed that the cardiohemodynamic and electrophysiological parameters in the *in vivo* experiments were stable > 2 h in the absence of pharmacological intervention (Sugiyama and Hashimoto, 1998; Usui et al., 1998; Satoh et al., 1999; Iwasaki et al., 2009).

### Experiment 3 (Exp. 3): Patch-Clamp Experiments

#### Cell Culture

Human embryonic kidney cell lines (HEK293 cells) stably co-expressing recombinant hKir3.1, hKir3.4 and hM2 (TOA EIYO LTD. Tokyo, Japan) were cultured in α-MEM supplemented with 10% fetal bovine serum (FBS), 12.5 μg/ml of G418 and 25 μg/ml of Zeocin. Chinese hamster ovary K1 (CHO-K1) cells stably expressing recombinant hK_V_1.5 (TOA EIYO LTD.) were cultured in Ham’s F12 medium supplemented with 10% FBS and 200 μg/ml of G418. CHO-K1 cells stably expressing recombinant hERG (B’SYS GmbH, Witterswill, Switzerland) were cultured in Ham’s F12 medium supplemented with 10% FBS, 100 μg/ml of G418, 100 μg/ml of hygromycin B and 2 mM of L-glutamine. CHO cells stably expressing recombinant hNa_V_1.5 (B’SYS) were cultured in Ham’s F12 medium supplemented with 10% FBS, 250 μg/ml of G418, 1 μg/ml of puromycin and 2 mM of L-glutamine. HEK293 cells stably co-expressing hCa_V_1.2 α, β_2_ and α_2_δ_1_ subunits (B’SYS) were cultured in D-MEM/Ham’s F12 medium supplemented with 10% FBS, 100 unit/mL of penicillin/streptomycin, 100 μg/ml of hygromycin B, 15 μg/ml of blasticidine S and 1 μg/ml of puromycin.

#### Patch-Clamp Recording

All ionic currents were recorded with automated whole-cell, patch-clamp technique. The inhibitory effects of 30–1000 μM of oseltamivir phosphate on Kir3.1/3.4 and K_V_1.5‐derived currents were assessed using port-a-patch automated patch-clamp technology (Nanion Technologies, Munich, Germany) at TOA EIYO LTD., whereas those of 10–1,000 μM on hERG, Na_V_1.5 and Ca_V_1.2-derived currents were evaluated using Qube 384 automated patch-clamp system (Sophion Bioscience A/S, Ballerup, Denmark) at Sophion Bioscience K.K. Each current was recorded at room temperature (20–25°C) except for Kir3.1/3.4-derived current which was done at 30°C. The recording data was analyzed by PatchMaster (ver. 2 × 69, HEKA Electronik GmbH, Lambrecht, Germany) for Kir3.1/3.4 and K_V_1.5-derived currents, or by Sophion Analyzer (ver. 6.5, Sophion Bioscience A/S) for hERG, Na_V_1.5 and Ca_V_1.2-derived ones. The cells for recording Kir3.1/3.4 or K_V_1.5-derived currents were exposed to oseltamivir phosphate solution in the order of rising concentration using a perfusion system. The cells for recording hERG, Na_V_1.5 or Ca_V_1.2-derived currents were exposed to the three test solutions; namely, vehicle, oseltamivir phosphate at either concentration of 10, 30, 100, 300 or 1,000 μM, and selective ionic channel blockers or sodium-free bath solution with an interval of the replacement time of > 14 min. The IC_50_ values were calculated by fitting the data to Hill equation using GraphPad prism 8 (ver. 8.31; GraphPad Software, Inc. CA, United States).

The protocol for recording Kir3.1/3.4-derived K^+^ current consisted of a 500 ms prepulse at –120 mV, followed by a 1,000 ms ramp pulse from –120 mV to + 60 mV with an interval of 10 s at a holding potential of −40 mV. The current amplitude was measured at + 60 mV for analyzing the concentration-response relationship (n = 3). The bath solution was composed of (in mM) 100 NaCl, 40 KCl, 1 MgCl_2_, 1.8 CaCl_2_, 10 D (+)-glucose and 10 HEPES at pH 7.4 with NaOH. The internal recording solution was composed of (in mM) 50 KCl, 10 NaCl, 60 KF, 2 MgCl_2_, 20 EGTA and 10 HEPES at pH 7.2 titrated with KOH. The K_V_1.5-derived K^+^ current was evoked by a 500 ms step pulse at 0 mV with an interval of 20 s at a holding potential of –70 mV. The peak amplitude was measured for analyzing the concentration-response relationship (n = 3). The external recording solution was composed of (in mM) 140 NaCl, 4 KCl, 1 MgCl_2_, 2 CaCl_2_, 5 D (+)-glucose and 10 HEPES at pH 7.4 titrated with NaOH, whereas the internal recording solution was the same as that used for recording Kir3.1/3.4 current. Tail current of hERG-derived K^+^ current was elicited by a 2,000 ms step pulse at –50 mV after a 1,000 ms prepulse at +40 mV with an interval of 30 s at a holding potential of −70 mV (n = 6). The peak amplitude of the tail currents was normalized with the following equation (I_peak_–I_b_)/(I_f_–I_b_); I_peak_ is the peak amplitude in the presence of oseltamivir; I_b_ is that with vehicle (1% PBS containing bath solution, basal); and I_f_ is that in the presence of 100 μM quinidine (fully blocked). The bath solution was composed of (in mM) 145 NaCl, 4 KCl, 2 CaCl_2_, 1 MgCl_2_, 10 D (+)-glucose and 10 HEPES at pH 7.4 titrated with NaOH. The internal recording solution was composed of (in mM) 120 KF, 20 KCl, 10 EGTA and 10 HEPES at pH 7.2 titrated with KOH. The Na_V_1.5‐derived Na^+^ current was evoked by a 20 ms step pulse at −10 mV every 10 s at a holding potential of −90 mV (n = 6). The peak amplitude in the presence of oseltamivir was measured and normalized using that with vehicle and that obtained in a sodium-free bath solution. To assess the frequency-dependent block of Na_V_1.5 by oseltamivir, a train of 30 step pulses at −10 mV for 20 ms at 1 Hz, 5 Hz or 10 Hz was applied, and the peak amplitude at the 30th test pulse (P30) was normalized by that at the first pulse (P1). The steady-state inactivation curves of Na_V_1.5 channels before and after application of vehicle and oseltamivir phosphate were obtained by the following protocol; a 500 ms prepulse ranging from –120 mV to –10 mV in 10 mV increments followed by a 20 ms test pulse at –10 mV with an interval of 20 s at a holding potential of –120 mV. The peak amplitude was normalized with the maximum peak amplitude for each cell in the same condition and was fitted with Boltzmann equation to calculate the half-maximum voltage of inactivation (V_1/2, inact_). The bath solution was composed of (in mM) 145 NaCl, 4 KCl, 2 CaCl_2_, 1 MgCl_2_, 10 D (+)-glucose and 10 HEPES at pH 7.4 titrated with NaOH. The sodium-free bath solution was composed of (in mM) 145 NMDG-Cl, 4 KCl, 2 CaCl_2_, 1 MgCl_2_, 10 D (+)-glucose and 10 HEPES at pH 7.4 titrated with NaOH. The internal recording solution was composed of (in mM) 10 NaCl, 140 CsF, 1 EGTA and 10 HEPES at pH 7.3 titrated with CsOH. The Ca_V_1.2-derived inward Ca^2+^ current was evoked by a 200 ms step pulse at 0 mV every 30 s at a holding potential of –80 mV (n = 6). The peak amplitude in the presence of oseltamivir was normalized using that with vehicle and that in the presence of 30 μM nifedipine. The bath solution was composed of (in mM) 145 NaCl, 4 KCl, 10 CaCl_2_ and 10 HEPES at pH 7.4 with NaOH. The internal recording solution was composed of (in mM) 112 CsCl, 2 NaCl, 28 CsF, 8.2 EGTA, 4 MgATP and 10 HEPES at pH 7.2 titrated with CsOH.

### Drugs

Oseltamivir phosphate (Tamiflu®, 75 mg capsule as oseltamivir, Chugai Pharmaceutical Co., Ltd. Tokyo, Japan) of six capsules were decapsulated, mixed with 15 ml of saline and sonicated for 30 min. The mixture was centrifuged at 1,200 × g for 30 min and its supernatant was treated with syringe filters of 0.45 μm pore size (EB-DISK 25, Kanto Chemical Co., Ltd. Tokyo, Japan). The solution containing 30 mg/ml of oseltamivir (MW: 312.41) was diluted to 3 and 0.3 mg/ml with saline for the *in vivo* experiment. Pilsicainide hydrochloride hydrate (MW: 317.85) (Sunrythm®, Daiichi-Sankyo Co., Ltd. Tokyo, Japan) was dissolved with saline in a concentration of 3 mg/ml, which was diluted to 1 mg/ml for the experiment. The dose of pilsicainide was calculated as its hydrochloride hydrate form. Oseltamivir phosphate (AK Scientific Inc. CA, United States) was dissolved in distilled water or PBS and diluted with each bath solution for patch clamp assay. The other drugs used were thiopental sodium (Ravonal®, Mitsubishi-Tanabe Pharma Co., Osaka, Japan), pentobarbital sodium (Tokyo Chemical Industry Co., Ltd. Tokyo, Japan), isoflurane (Pfizer Japan Inc. Tokyo, Japan) and halothane (Fluothane®, Takeda Pharmaceutical Co., Ltd. Osaka, Japan).

### Statistical Analysis

Data are presented as mean ± S.E.M. Differences within a parameter were evaluated with one-way, repeated-measures analysis of variance (ANOVA) followed by Contrasts as a post hoc-test for mean values comparison, whereas those between the groups were assessed by unpaired *t*-test. A *p* value < 0.05 was considered to be significant, and those among the groups were done by one-way, factorial ANOVA followed by Fisher’s LSD test.

## Results

There was no animal excluded from cardioversion experiments (Exp. 1) or electrophysiological studies (Exp. 2) after its randomization. No episode of lethal ventricular arrhythmias or hemodynamic collapse was observed during cardioversion experiments (Exp. 1) or electrophysiological studies (Exp. 2).

### Cardioversion Experiments (Exp. 1)

**FIGURE 1:**
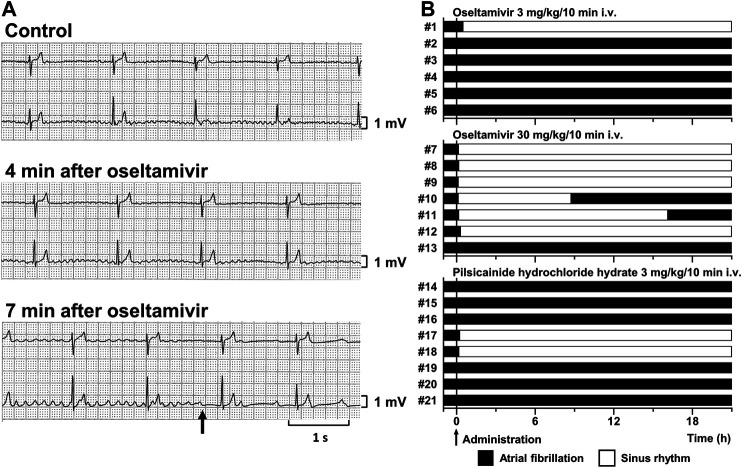
Antiarrhythmic effects of oseltamivir and pilsicainide hydrochloride hydrate on the conscious dogs with persistent AF. **(A)** Typical tracings of Holter electrocardiogram (NASA lead: **upper**; and CM5 lead: **lower**) before (Control; **top**), 4 min (4 min after oseltamivir; **middle**) and 7 min (7 min after oseltamivir; **bottom**) after the start of intravenous administration of 30 mg/kg/10 min of oseltamivir (animal number #9). The AF was terminated at 7 min (arrow). **(B)** Time courses of the antiarrhythmic effects of oseltamivir in doses of 3 (**top**, n = 6) and 30 mg/kg/10 min i.v. (**middle**, n = 7) and pilsicainide hydrochloride hydrate in a dose of 3 mg/kg/10 min i.v. (**bottom**, n = 8).

Typical tracings of Holter electrocardiogram before and during the intravenous infusion of oseltamivir are depicted in Figure 1A, whereas time courses of anti-AF effects of oseltamivir and pilsicainide are summarized in Figure 1B. Three mg/kg of oseltamivir terminated the AF in 1 out of 6 animals at 30 min after the start of administration. Its 30 mg/kg did it in 6 out of 7 animals at 8 (#7), 12 (#8), 7 (#9), 6 (#10), 9 (#11) and 19 min (#12) after the start of administration. The AF recurred in 2 animals at 8.6 (#10) and 16 h (#11) after the termination of AF. Meanwhile, 3 mg/kg of pilsicainide hydrochloride hydrate terminated the AF in 2 out of 8 animals at 15 (#17) and 11 min (#18) after the start of administration.

### Electrophysiological Studies (Exp. 2)

**FIGURE 2:**
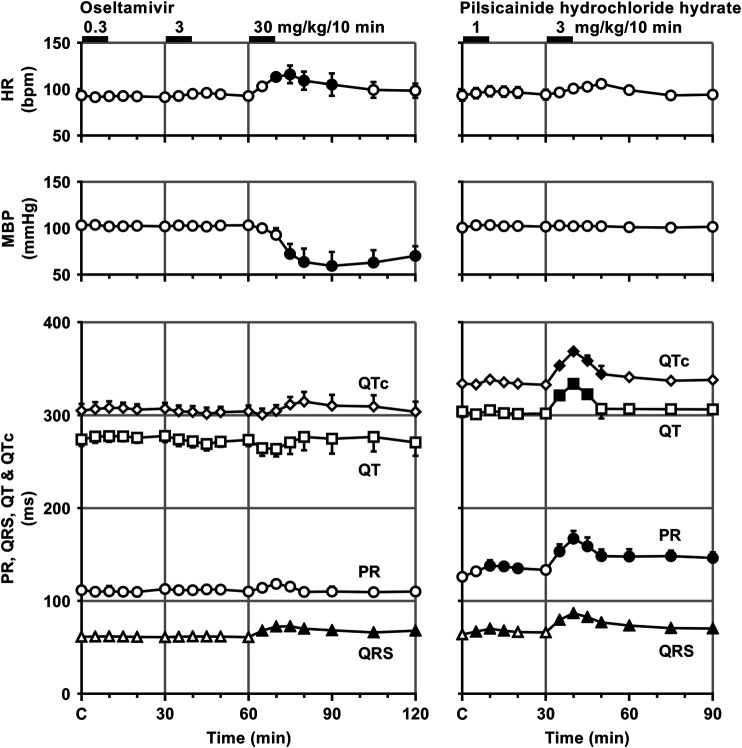
Time courses of changes in the heart rate (HR), mean blood pressure (MBP), PR interval (PR), QRS width (QRS), QT interval (QT) and QTc after the administrations of oseltamivir **(left)** and pilsicainide hydrochloride hydrate **(right)**. QT interval was corrected with Van de Water’s formula: QTc = QT−0.087 × (RR−1,000) with RR given in ms (Van de Water et al., 1989). Data are presented as mean ± S.E.M. (n = 4 for each drug). Closed symbols represent significant differences from their corresponding pre-drug basal control value (C) by *p* < 0.05.

**FIGURE 3:**
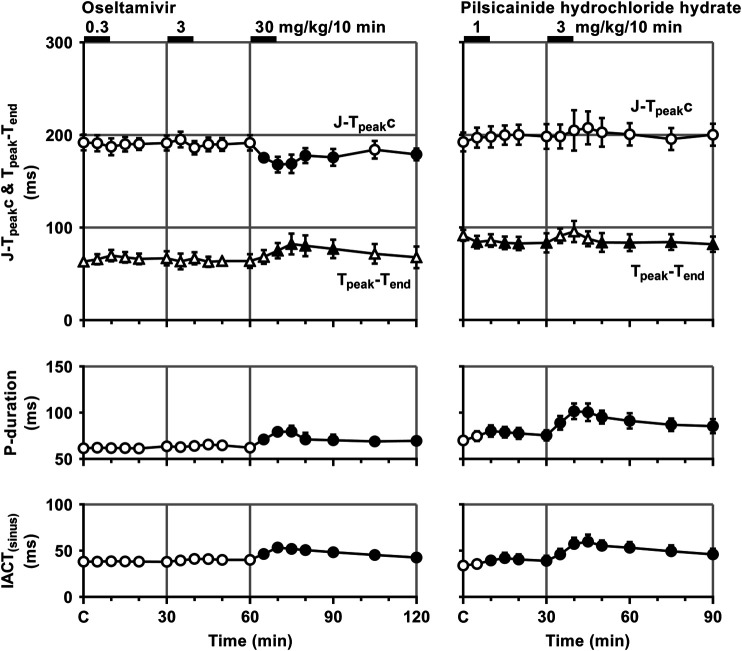
Time courses of changes in the J-T_peak_c, T_peak_-T_end_, P-wave duration (P-duration) and inter-atrial conduction time (IACT) during sinus rhythm (IACT_(sinus)_) after the administrations of oseltamivir **(left)** and pilsicainide hydrochloride hydrate **(right)**. J-T_peak_ was corrected by the following formula: J-T_peak_c = J-T_peak_/RR^0.58^ with RR given in seconds (Johannesen et al., 2014). Data are presented as mean ± S.E.M. (n = 4 for each drug). Closed symbols represent significant differences from their corresponding pre-drug basal control value (C) by *p* < 0.05.

Time courses of changes in the variables during sinus rhythm after the administration of oseltamivir and pilsicainide are summarized in Figures 2, 3. The pre-drug basal control values (C) of the heart rate, mean blood pressure, PR interval, QRS width, QT interval, QTc, J-T_peak_c, T_peak_-T_end_, P-duration and IACT_(sinus)_ were 93 ± 1 beats/min, 103 ± 5 mmHg, 112 ± 5 ms, 61 ± 3 ms, 274 ± 7 ms, 305 ± 7, 192 ± 9, 63 ± 5 ms, 62 ± 3 ms and 38 ± 2 ms in the oseltamivir-treated animals (n = 4), whereas those were 93 ± 6 beats/min, 101 ± 3 mmHg, 126 ± 3 ms, 64 ± 2 ms, 304 ± 6 ms, 334 ± 5, 192 ± 10, 91 ± 6 ms, 70 ± 5 ms and 34 ± 4 ms in the pilsicainide-treated animals (n = 4), respectively. There was no significant difference in these values between the groups except for PR interval, QT interval, QTc or T_peak_-T_end_.

The low and middle doses of oseltamivir hardly altered any of the variables during sinus rhythm. The high dose increased the heart rate for 10–30 min, but decreased the mean blood pressure for 15–60 min. It also prolonged the QRS width, P-duration and IACT_(sinus)_ for 5–60 min and T_peak_-T_end_ for 10–30 min, but shortened the J-T_peak_c for 5–30 min and at 60 min, whereas no significant change was detected in the PR interval, QT interval or QTc. Meanwhile, the low dose of pilsicainide prolonged the PR interval for 10–20 min, QRS width for 5–15 min and P-duration and IACT_(sinus)_ for 10–30 min, but shortened the T_peak_-T_end_ at 5 min and for 15–30 min, whereas no significant change was detected in the other variables. The high dose prolonged the PR interval, QRS width, P-duration and IACT_(sinus)_ for 5–60 min, QT interval for 5–15 min and QTc for 5–20 min, but shortened the T_peak_-T_end_ for 20–60 min, whereas no significant change was detected in the other variables.

**FIGURE 4:**
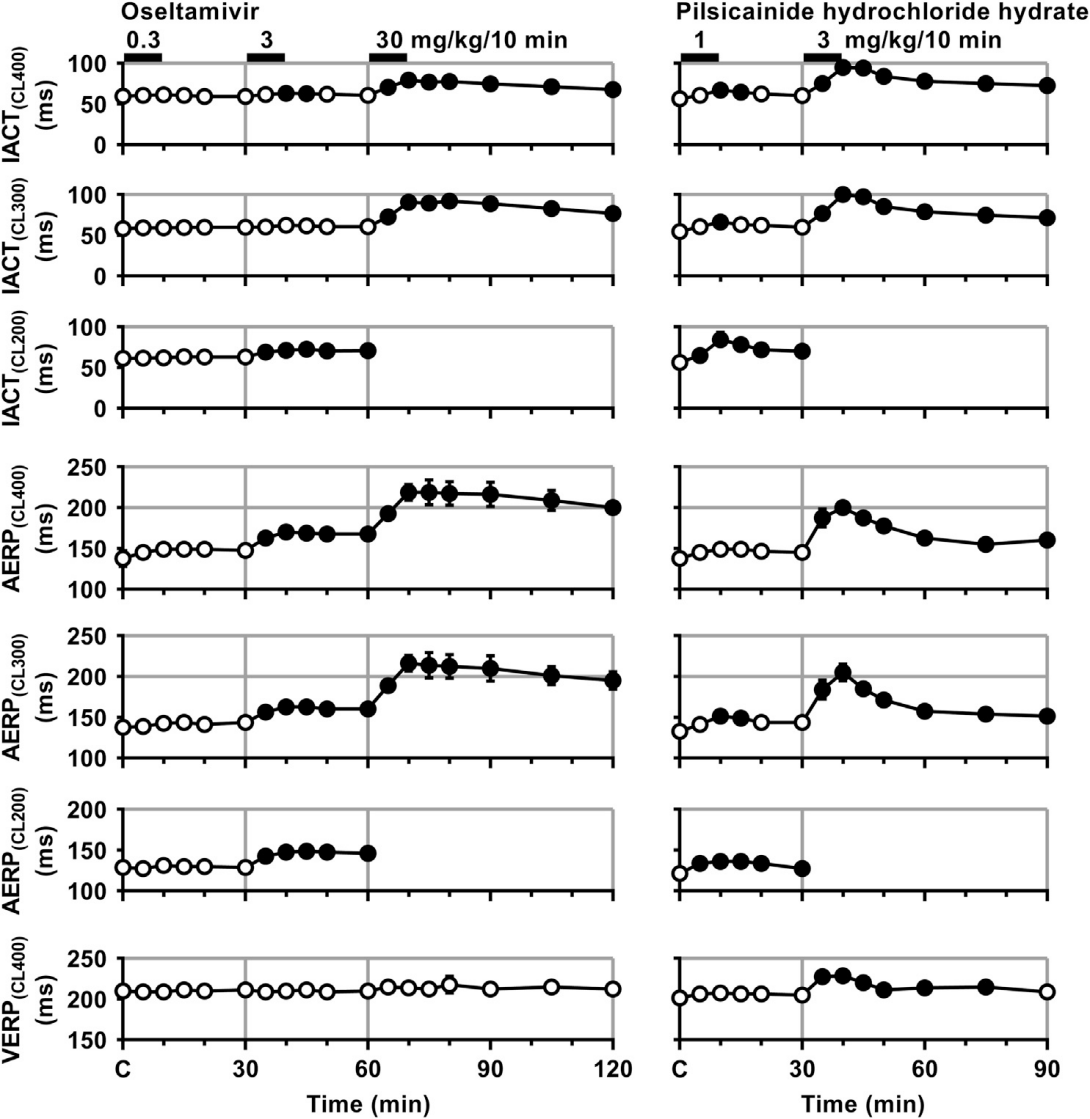
Time courses of changes in the inter-atrial conduction time (IACT) at pacing cycle lengths of 400 ms (IACT_(CL400)_), 300 ms (IACT_(CL300)_) and 200 ms (IACT_(CL200)_); atrial effective refractory period (AERP) at basic pacing cycle lengths of 400 ms (AERP_(CL400)_), 300 ms (AERP_(CL300)_) and 200 ms (AERP_(CL200)_); and ventricular effective refractory period (VERP) at a basic pacing cycle length of 400 ms (VERP_(CL400)_) after the administrations of oseltamivir **(left)** and pilsicainide hydrochloride hydrate **(right)**. Data are presented as mean ± S.E.M. (n = 4 for each drug). Closed symbols represent significant differences from their corresponding pre-drug basal control value (C) by *p* < 0.05.

**FIGURE 5:**
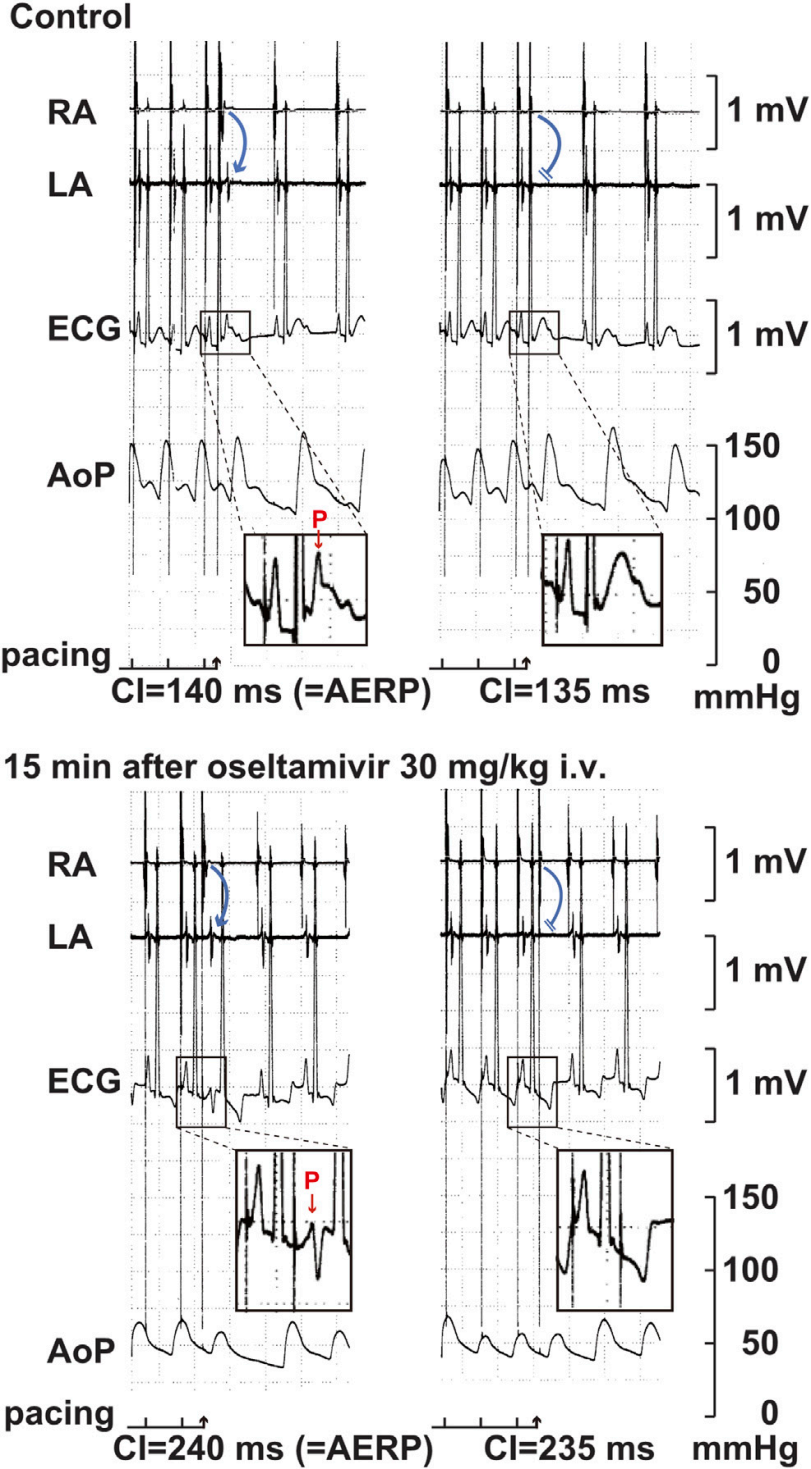
Typical tracings of the right (RA) as well as left (LA) atrial electrograms, electrocardiogram (ECG) and aortic pressure (AoP) during the atrial programed electrical stimulation for the assessment of atrial effective refractory period (AERP) at a basic pacing cycle length of 400 ms at pre-drug control (Control; **upper panels**) and 15 min after the start of intravenous administration of 30 mg/kg of oseltamivir (15 min after oseltamivir 30 mg/kg i.v.; **lower panels**). A premature right atrial stimulation with a coupling interval (CI) of 140 ms induced electrical activity in LA electrogram (blue arrow) and *p* wave in ECG (red arrow) **(upper left)**, whereas that of 135 ms failed to develop either of them **(upper right)**, indicating that the AERP was 140 ms. After the administration oseltamivir, a premature right atrial stimulation with a CI of 240 ms induced the electrical activity and *p* wave **(lower left)**, whereas that of 235 ms did not develop either of them **(lower right)**, indicating that the AERP was 240 ms. Administration of 30 mg/kg of oseltamivir decreased the aortic pressure. Moreover, the programmed atrial stimulation-induced atrial tachycardia followed by atrial premature contraction could have enhanced the hypotensive action.

Time courses of changes in the inter-atrial conduction time and atrial as well as ventricular effective refractory period after the administration of oseltamivir and pilsicainide are summarized in Figure 4. Typical tracings of the right as well as left atrial electrogram, electrocardiogram and aortic pressure during the atrial programmed electrical stimulation for the assessment of AERP_(CL400)_ are depicted in Figure 5. The pre-drug basal control values (C) of the IACT_(CL400)_, IACT_(CL300)_, IACT_(CL200)_, AERP_(CL400)_, AERP_(CL300)_, AERP_(CL200)_ and VERP_(CL400)_ were 60 ± 6, 58 ± 6, 61 ± 6, 138 ± 10, 138 ± 6, 129 ± 7 and 210 ± 5 ms in the oseltamivir-treated animals (n = 4), whereas those were 56 ± 5, 54 ± 4, 56 ± 4, 138 ± 5, 133 ± 3, 121 ± 6 and 201 ± 5 ms in the pilsicainide-treated animals (n = 4), respectively. There was no significant difference in these values between the groups.

The low dose of oseltamivir hardly altered any of the variables. The middle dose prolonged the IACT_(CL400)_ for 10–15 min and IACT_(CL200)_ for 5–30 min; and AERP_(CL400)_, AERP_(CL300)_ and AERP_(CL200)_ for 5–30 min; whereas no significant change was detected in the IACT_(CL300)_ or VERP_(CL400)_. The high dose prolonged the IACT_(CL400)_ and IACT_(CL300)_ for 5–60 min; and AERP_(CL400)_ and AERP_(CL300)_ for 5–60 min; whereas no significant change was detected in VERP_(CL400)_. Meanwhile, the low dose of pilsicainide prolonged the IACT_(CL400)_ for 10–15 min, IACT_(CL300)_ at 10 min, IACT_(CL200)_ for 5–30 min; AERP_(CL300)_ for 10–15 min and AERP_(CL200)_ for 5–30 min, whereas no significant change was detected in the AERP_(CL400)_ or VERP_(CL400)_. The high dose prolonged the IACT_(CL400)_ and IACT_(CL300)_ for 5–60 min; AERP_(CL400)_ and AERP_(CL300)_ for 5–60 min; and VERP_(CL400)_ for 5–45 min. Since the atrium could not be paced at a cycle length of 200 ms after the administration of the high dose of both drugs due to the prolongation of AERP to > 200 ms, we could not obtain the IACT_(CL200)_ or AERP_(CL200)_ for 5–60 min.

### Patch-Clamp Experiments (Exp. 3)

**FIGURE 6:**
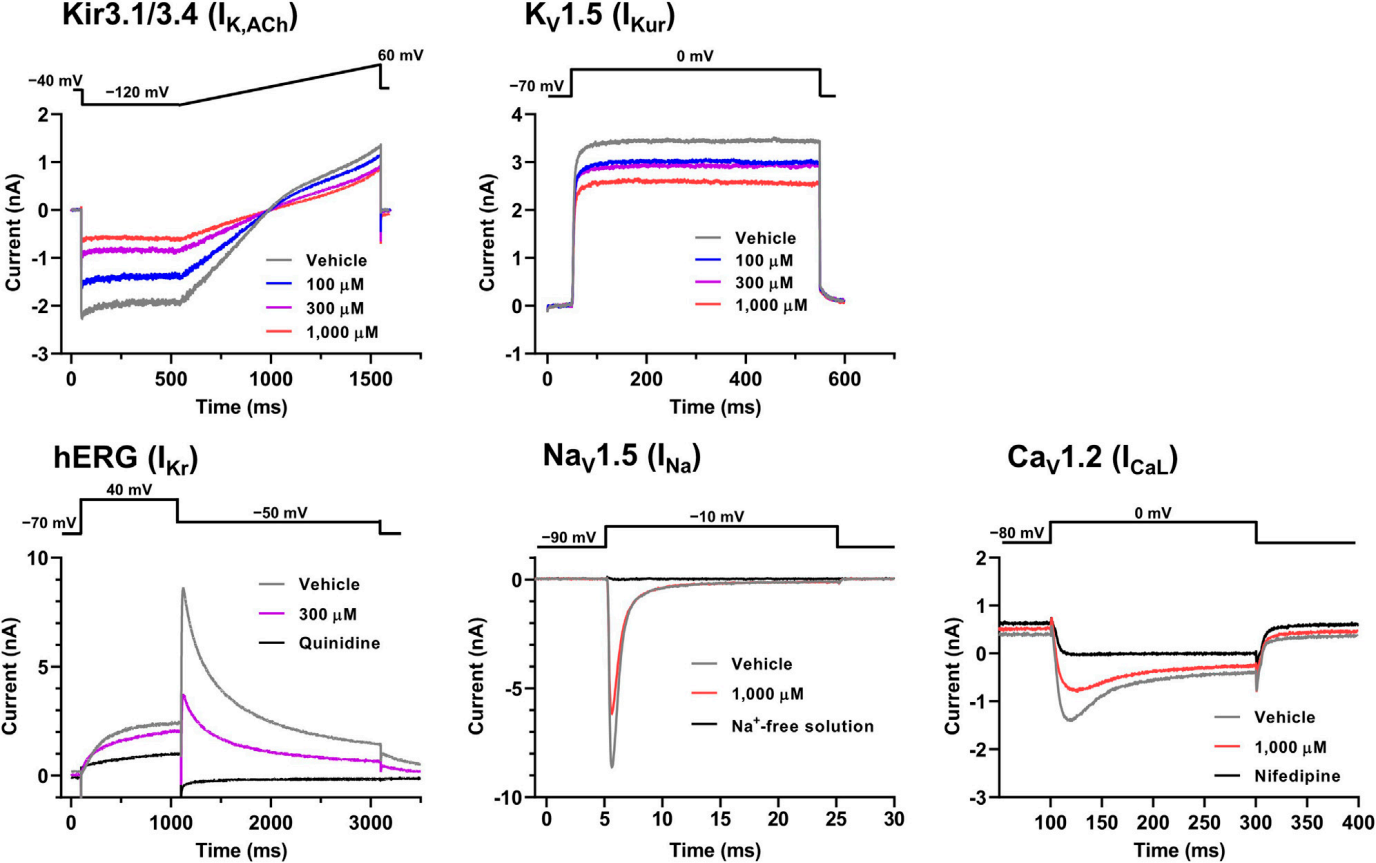
Voltage protocols **(upper)** and representative current waveforms **(lower)** showing the inhibitory actions of oseltamivir phosphate on Kir3.1/3.4, K_V_1.5, hERG, Na_V_1.5 and Ca_V_1.2-derived currents. The current waveforms of Kir3.1/3.4 and K_V_1.5-derived currents were recorded with vehicle and 100–1,000 μM of oseltamivir, whereas those of hERG, Na_V_1.5, Ca_V_1.2-derived currents were done with vehicle, 300 or 1,000 μM oseltamivir, and relevant ion channel blockers (100 μM quinidine or 30 μM nifedipine) or Na^+^-free solution.

**FIGURE 7:**
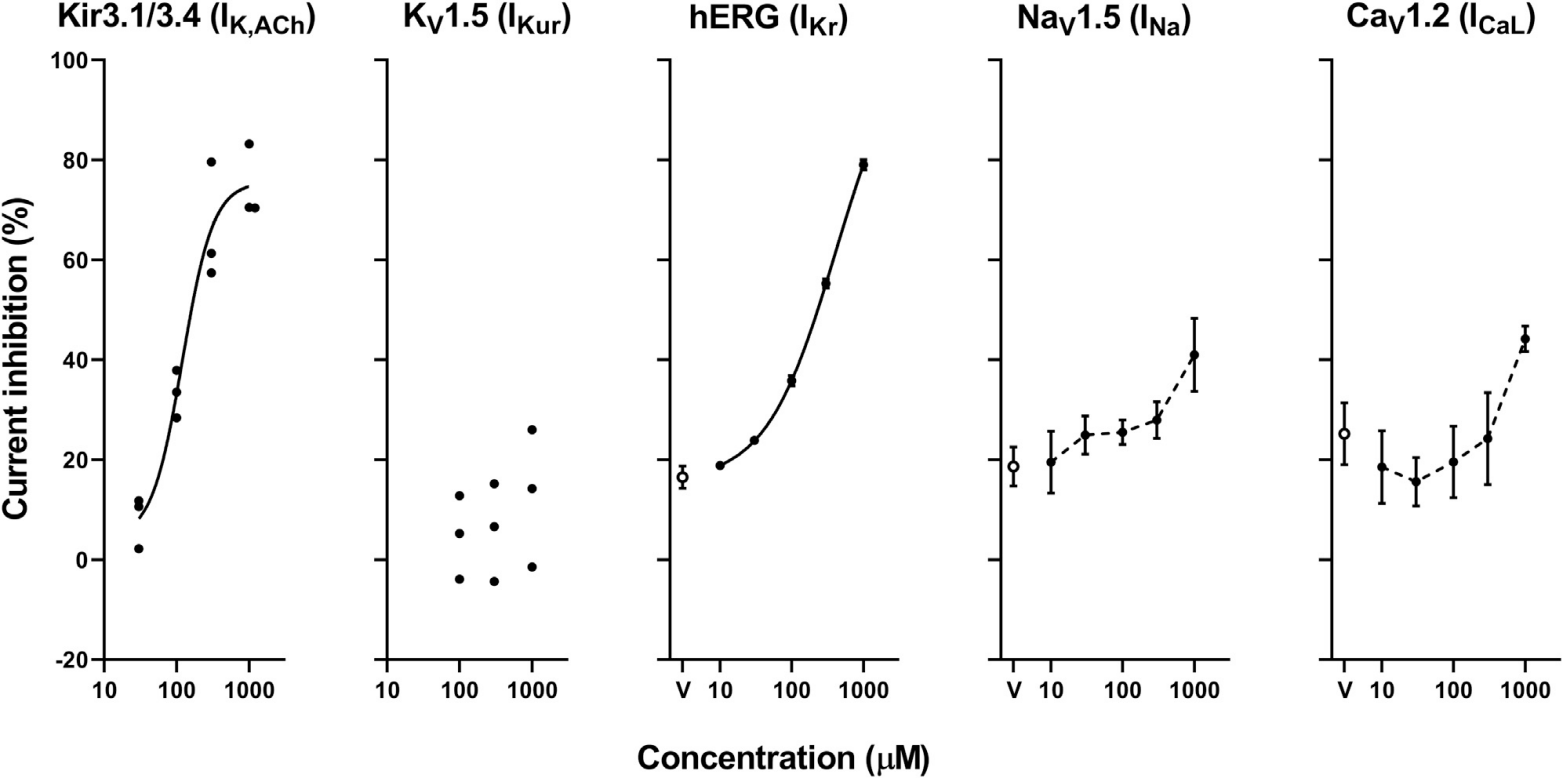
Concentration-response curves showing the inhibitory action (%) of oseltamivir phosphate or vehicle (V) on Kir3.1/3.4, K_V_1.5, hERG, Na_V_1.5 and Ca_V_1.2-derived currents. Data are presented as individual data (n = 3 for Kir3.1/3.4 and K_V_1.5-derived currents) or mean ± S.E.M. (n = 6 for hERG, Na_V_1.5 and Ca_V_1.2-derived currents). The concentration-response curves of Kir3.1/3.4 and hERG-derived currents were fitted with Hill equation with solid line and their IC_50_ values were 160 and 231 μM, respectively, whereas those of Na_V_1.5 and Ca_V_1.2-derived currents were made by interpolating those values of current inhibition at each concentration with dashed line.

**FIGURE 8:**
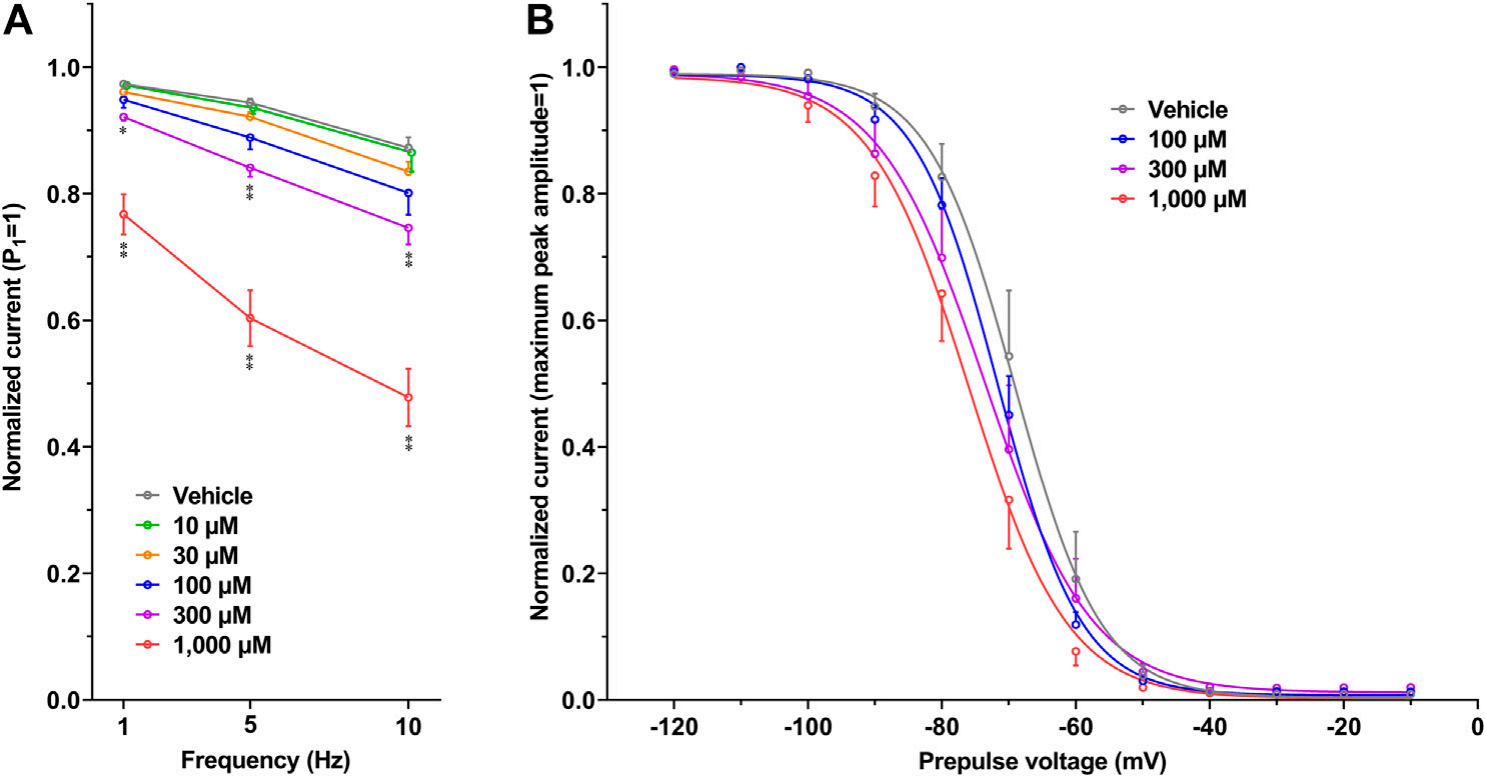
Frequency- and Voltage-dependent block of Na_V_1.5 by oseltamivir. **(A)** The normalized peak amplitudes of Na_V_1.5-derived current at 30th test pulse in the presence of vehicle (n = 5) and oseltamivir (n = 6 for each concentration) were compared at the pulse frequencies of 1, 5 and 10 Hz. The peak amplitude was normalized by that at 1st step pulse (P1 = 1). The reduction of Na_V_1.5 current by oseltamivir was augmented in a frequency-dependent manner. *p < 0.05 and **p < 0.01 vs. vehicle at the same frequency. **(B)** The steady-state inactivation curves of Na_V_1.5 channels in the presence of vehicle (n = 6) and oseltamivir (n = 6 for each concentration). The peak amplitude was normalized by the maximum peak amplitude (maximum peak amplitude = 1). Oseltamivir negatively shifted the curves in a concentration-related manner. Data are presented as mean ± S.E.M.

The representative current waveforms showing the inhibitory actions of oseltamivir on Kir3.1/3.4 (I_K,ACh_), K_V_1.5 (I_Kur_), hERG (I_Kr_), Na_V_1.5 (I_Na_) and Ca_V_1.2-derived currents (I_CaL_) along with voltage pulse protocols are shown in Figure 6, whereas its concentration-response curves showing its inhibitory action (%) on these currents are summarized in Figure 7. Oseltamivir suppressed each of the currents in a concentration-related manner. The IC_50_ values for Kir3.1/3.4 and hERG-derived currents were 160 and 231 μM, respectively. Meanwhile, oseltamivir in a concentration of 1,000 µM inhibited the K_V_1.5, Na_V_1.5 and Ca_V_1.2‐derived currents by 13 ± 8, 22 ± 9 and 19 ± 6%, respectively. Frequency- and voltage-dependent properties of I_Na_ block by oseltamivir were further analyzed, which are summarized in Figure 8 and Table 1. Oseltamivir enhanced its I_Na_ block in a frequency-dependent manner, and achieved significant differences at 1, 5 and 10 Hz when the concentrations were 300 (Δnormalized current: −0.052, −0.103 and −0.127) and 1,000 µM (Δnormalized current: −0.206, −0.340 and −0.394, respectively) (Figure 8A). Meanwhile, oseltamivir negatively shifted the steady-state inactivation curve of Na_V_1.5 channels in a concentration-related manner (Figure 8B), and achieved significant differences in V_1/2, inact_ when the concentrations were 300 and 1,000 μM (Table 1). Oseltamivir tended to suppress the I_Na_ at prepulse voltage of −90, −80 and −70 mV when the concentrations were 100 (Δnormalized current: −0.021, −0.045 and −0.093), 300 (Δnormalized current: −0.075, −0.128 and −0.147) and 1,000 (Δnormalized current: −0.109, −0.184 and −0.227, respectively).

**TABLE 1:** Effect of oseltamivir on the steady-state inactivation curve of Na_V_1.5.

Concentration (μM)	10	30	100	300	1,000
ΔΔV_1/2, inact_ (mV)	−0.1 ± 1.2	0.0 ± 1.3	−1.7 ± 1.2	−4.1 ± 1.2*	−5.2 ± 1.4*

The half-maximum voltage of steady-state inactivation (V_1/2, inact_) was calculated by using Boltzmann equation for each cell. The differences of V_1/2, inact_ between pre- and post-applications of either vehicle or oseltamivir were calculated to obtain ΔV_1/2, inact_. The differences in the ΔV_1/2, inact_ between oseltamivir– and vehicle-treatments were done to provide ΔΔV_1/2, inact_ (n = 6 for each concentration). Data are presented as mean ± S.E.M. *p < 0.05 vs. vehicle.

## Discussion

### Rationale of Drug Doses

In our previous study using the halothane-anesthetized dogs, 3 and 30 mg/kg, i.v. of oseltamivir prolonged the P-wave duration in a dose-related manner, reflecting its inhibitory action on I_Na_ (Kitahara et al., 2013), which is known to play an important role in the anti-AF effect (Harvey and Grant, 2018). Thus, we selected those doses for cardioversion experiments (Exp. 1). In order to better characterize effects of oseltamivir on the atrial electrophysiological variables in electrophysiological studies (Exp. 2), we adopted the same protocol as that used in the standard electropharmacological study (Kitahara et al., 2013). Since 0.3, 3 and 30 mg/kg, i.v. over 10 min of oseltamivir provided peak plasma concentrations of 1.2 (3.8), 10.6 (33.9) and 117.5 (376.1) μg/ml (μM), respectively in our previous study and its protein binding ratio was < 50% (Kitahara et al., 2013), their free plasma concentrations could be estimated to be > 2, > 17 and > 188 μM, respectively. Accordingly, we chose the concentrations of 10–1,000 μM of oseltamivir for patch-clamp experiments (Exp. 3). On the other hand, pilsicainide hydrochloride hydrate has been clinically used in an intravenous dose of 1 mg/kg over 10 min, providing peak plasma concentration of 1.74 μg/ml in healthy human subjects according to the interview form from manufacturer. Thus, we assessed 3 mg/kg, i.v. over 10 min of pilsicainide hydrochloride hydrate in cardioversion experiments (Exp. 1) to better detect its anti-AF action, and its 1 and 3 mg/kg, i.v. over 10 min in electrophysiological studies (Exp. 2).

### Antiarrhythmic Effects on the Persistent AF

In order to better characterize the canine model of persistent AF, in this study we calibrated it using anti-AF drug pilsicainide (Plosker, 2010). Three mg/kg of pilsicainide hydrochloride hydrate terminated AF in 2 out of 8 animals, resulting in sinus rhythm (25%). Meanwhile, 3 and 30 mg/kg of oseltamivir did it in 1 out of 6 (17%) and 6 out of 7 animals (86%), respectively, indicating its potent anti-AF efficacy. It should be noted that AF recurred in 2 animals after the high dose of oseltamivir, reflecting a causal relationship between the plasma concentration of oseltamivir and its anti-AF action. Since we previously reported that the time course of the plasma concentration of oseltamivir followed a pattern that was predicted by the two-compartment theory of pharmacokinetics in the halothane-anesthetized dog (Kitahara et al., 2013), the recurrence of AF in 2 animals may be associated with the decrease of the plasma concentration of oseltamivir. In addition, lethal ventricular arrhythmia or hemodynamic collapse was not induced during the experimental period, indicating favorable safety pharmacological profile of oseltamivir, which may confirm previously described cardiovascular safety profile (Nakamura et al., 2016). Thus, oseltamivir may become a promising seed compound to develop efficacious and safe anti-AF drugs.

### *In vivo* Electropharmacological Effects

The mechanisms of anti-AF effects of oseltamivir were analyzed using the anesthetized dogs with the intact hearts. In our previous study, 30 mg/kg of oseltamivir decreased the mean blood pressure by 7 mmHg possibly through I_Na_ and/or I_CaL_ inhibitions along with the heart rate reduction (−9 bpm) (Kitahara et al., 2013). Meanwhile in this study, the same dose of oseltamivir decreased the mean blood pressure by 44 mmHg, but increased the heart rate by 23 bpm, which may indicate occurrence of the reflex-mediated increase of sympathetic tone. The larger depressor action in the currently used animals than the previous ones might be partly associated with differences in their breeding companies (TOYO Beagle vs. Iar:Beagle) and/or ages (2 years vs. 12 months). This difference needs further analysis; however, such depressor action can be expected to be smaller in conscious state.

In the previous study (Kitahara et al., 2013), the same doses of oseltamivir as used in this study delayed the atrioventricular conduction and repolarization in a dose-related manner possibly through direct I_CaL_ and I_Kr_ inhibition, respectively (Takahara et al., 2013). In this study, however, they tended to prolong these variables, which did not achieve a statistical significance, since the increase of sympathetic tone could have counteracted the actions of oseltamivir by indirectly enhancing I_CaL_ and I_Ks_ (Harvey and Grant, 2018). The high dose of oseltamivir delayed the intra-atrial and intra-ventricular conductions by 18 and 11 ms, whereas that of pilsicainide did the variables by 32 and 23 ms, respectively, showing the higher atrial selectivity of oseltamivir than that of pilsicainide. Importantly, their increments were 16 and 8 ms for oseltamivir, respectively in the previous study (Kitahara et al., 2013), indicating that sympathetic tone would have hardly modified these effects.

There is a discrepancy between the significant I_Kr_-blocking effect of oseltamivir *in vitro* as discussed below and no change in ventricular refractory period or QTc interval *in vivo*. In order to better understand these findings, we analyzed the early (J-T_peak_c) and late (T_peak_-T_end_) ventricular repolarization periods of electrocardiogram; the former could reflect the net balance between inward I_CaL_ and outward I_Ks_ plus I_Kr_, and the latter would indicate the extent of I_Kr_ modification along with transmural dispersion of the ventricular repolarization period. As shown in Figure 3, oseltamivir significantly prolonged the T_peak_-T_end_, indicating its I_Kr_ blocking action *in vivo*, which was in accordance with that demonstrated in patch-clamp experiments (Exp. 3). More importantly, oseltamivir significantly shortened the J-T_peak_c possibly through direct I_CaL_ suppression and/or indirect I_Ks_ enhancement induced by the hypotension-induced, reflex-mediated, increase of sympathetic tone, which may have counteracted the I_Kr_ blockade-dependent J-T_peak_c and QT/QTc prolongation.

In order to better understand how oseltamivir terminated the AF, we assessed its effects on the IACT and AERP; surrogate markers for anti-AF action (Kambayashi et al., 2020), which was compared with those of pilsicainide as summarized in Table 2. The middle and high doses of oseltamivir increased the IACT in a frequency-dependent manner but prolonged the AERP in a reverse frequency-dependent manner, suggesting that the former may be related to I_Na_ inhibition and that the latter would be more associated with I_Kr_ inhibition than I_Na_ suppression. Meanwhile, pilsicainide prolonged the IACT and AERP in a frequency-dependent manner except for AERP_(CL200)_, indicating that I_Na_ inhibition may play an important role for both of them. It should be noted that the high dose of oseltamivir prolonged the AERP greater than that of pilsicainide, whereas its reverse was true for the IACT as shown in Table 2. The anti-AF action of the high dose of oseltamivir may more depend on the prolongation of AERP than IACT. Taking the result of cardioversion experiments (Exp. 1) into account, the AERP may be more sensitive marker than the IACT for predicting antiarrhythmic effects against the persistent AF. In addition, the high dose of oseltamivir prolonged the AERP_(CL400)_ and VERP_(CL400)_ by 81 and 4 ms (ΔAERP/ΔVERP = 20.3), whereas that of pilsicainide did the variables by 63 and 28 ms (ΔAERP/ΔVERP = 2.3), respectively, indicating that oseltamivir may have much higher atrial selectivity than pilsicainide, which was shown in Table 2.

**TABLE 2:** Electrophysiological effects on the inter-atrial conduction and effective refractory period.

Drug	Oseltamivir		Pilsicainide hydrochloride hydrate	
Dose (mg/kg/10 min, i.v.)	3	30	1	3
IACT_(CL400)_ (ms)	+3	+20	+11	+38
IACT_(CL300)_ (ms)	+4	+32	+12	+46
IACT_(CL200)_ (ms)	+10	N/A	+28	N/A
AERP_(CL400)_ (ms)	+33	+81	+11	+63
AERP_(CL300)_ (ms)	+25	+79	+19	+73
AERP_(CL200)_ (ms)	+19	N/A	+15	N/A
VERP_(CL400)_ (ms)	0	+4	+6	+28
Atrial selectivity	∞	20.3	1.8	2.3

Each value represents the change from its corresponding pre-drug control value at 10 min after the start of intravenous administration. Atrial selectivity was calculated as a ratio of mean ΔAERP(CL400) to mean ΔVERP(CL400). IACT: Inter-atrial conduction time; AERP: Atrial effective refractory period; VERP: Ventricular effective refractory period; CL: pacing cycle length; and N/A: not available due to prolongation of AERP > 200 ms.

### *In vitro* Electropharmacological Effects

The patch clamp assay indicated that oseltamivir has multi-channel blocking action and suppressed the currents in the order of I_K,ACh_ > I_Kr_ >> I_Na_ > I_CaL_ > I_Kur_, of which IC_50_ values of I_K,ACh_ and I_Kr_ were 160 and 231 μM, respectively. The estimated free peak plasma concentration of oseltamivir would be > 188 μM after its high dose administration in cardioversion experiments (Exp. 1) and electrophysiological studies (Exp. 2), indicating that oseltamivir may have potently inhibited I_K,ACh_ and moderately suppressed I_Kr_ in those experiments. Although I_K,ACh_ as well as I_Kur_ has been shown to be predominantly expressed in the atrium (Gaborit et al., 2007), I_K,ACh_ and I_Kur_ inhibitors have been clinically shown to have a limited efficacy against AF (Heijman et al., 2017; Camm et al., 2019; Peyronnet and Ravens, 2019), implying the limitation of selective atrial ionic channel inhibitors. Importantly, the I_K,ACh_ suppression would depolarize the resting potential of atrial myocytes, which may result in a delay of Na^+^ channel recovery and an increase of inactivated Na^+^ channels (Yamada et al., 1998; Harvey and Grant, 2018). Similar synergic effect of I_Na_ and I_Kr_ inhibition has been shown in dogs (Aguilar et al., 2015). As shown in patch-clamp experiments (Exp. 3), oseltamivir blocked I_Na_ in a frequency-dependent manner, whereas it negatively shifted the steady-state inactivation curve of Na_V_1.5 in a concentration-related manner. These results can partly explain why oseltamivir inhibited the conduction of the atria *in vivo* at atrial beating rate of 100–150 bpm in electrophysiological studies (Exp. 2), in which resting membrane potential could be around −80 mV (Pandit, 2018). Moreover, constitutively active I_K,ACh_ in the atrium under chronic AF has been reported to increase, which may contribute to the shortening of the AERP that will promote the onset of reentry leading to persistence of AF (Dobrev et al., 2005; Heijman et al., 2017). Thus, I_K,ACh_ inhibitory action of oseltamivir may play a pivotal role in terminating the persistent AF, which was confirmed in cardioversion experiments (Exp. 1). In addition, I_K,ACh_ inhibition in addition to I_Kr_ suppression by oseltamivir may have prolonged AERP, also contributing to its anti-AF.

### Clinical Implication and Study Limitation

The estimated peak plasma concentrations of oseltamivir in cardioversion experiments (Exp. 1) and electrophysiological studies (Exp. 2) would be 100–1,000 and 10–1,000 times higher than that attained after its clinically recommended oral dose against influenza virus infection which was 115 ng/ml in healthy human subjects (Kitahara et al., 2013). Although oseltamivir could be well tolerated in the dogs having persistent AF beside the chronic atrioventricular conduction block, a frequent use of this drug for AF in clinical may increase the possibility to induce oseltamivir–resistant influenza virus. Thus, daily use of oseltamivir for patient having persistent AF should not be recommended for protecting the public health. Oseltamivir is metabolized extensively and predominantly by hepatic carboxylesterase to its sole active metabolite showing anti-influenza virus action (McClellan and Perry, 2001) which lacks electrophysiological activity on the heart (Fukushima et al., 2015). Developing a novel formulation that can avoid the hepatic first-pass effect and/or modulating its chemical structure that will be resistant to carboxylesterase would be able to reduce the effective dose against AF, and may help facilitate the development of ideal anti-AF drugs.

## Conclusion

Oseltamivir can exert a powerful anti-AF action through its ideal multi-channel blocking effects on I_K,ACh_, I_Kr_ and I_Na_. Although developing a novel formulation that can reduce the effective dose against AF may be mandatory, oseltamivir would be a promising seed compound for developing efficacious and safe anti-AF drugs.

## Data Availability

The raw data supporting the conclusions of this article will be made available by the authors, without undue reservation, to any qualified researcher.
